# Delivery of *E. coli* Nissle to the mouse gut by mucoadhesive microcontainers does not improve its competitive ability against strains linked to ulcerative colitis

**DOI:** 10.1093/femsle/fnad110

**Published:** 2023-10-20

**Authors:** Pi Westi Bondegaard, Anders Meyer Torp, Priscila Guerra, Katja Ann Kristensen, Juliane Fjelrad Christfort, Karen Angeliki Krogfelt, Line Hagner Nielsen, Kinga Zor, Anja Boisen, Martin Steen Mortensen, Martin Iain Bahl, Tine Rask Licht

**Affiliations:** National Food Institute, Technical University of Denmark, Kgs. Lyngby, 2800, Denmark; National Food Institute, Technical University of Denmark, Kgs. Lyngby, 2800, Denmark; National Food Institute, Technical University of Denmark, Kgs. Lyngby, 2800, Denmark; National Food Institute, Technical University of Denmark, Kgs. Lyngby, 2800, Denmark; Department of Health Technology, Technical University of Denmark, Kgs. Lyngby, 2800, Denmark; Department of Science and Environment, Molecular and Medical Biology, Roskilde University, Roskilde, 4000, Denmark; Department of Health Technology, Technical University of Denmark, Kgs. Lyngby, 2800, Denmark; Department of Health Technology, Technical University of Denmark, Kgs. Lyngby, 2800, Denmark; Department of Health Technology, Technical University of Denmark, Kgs. Lyngby, 2800, Denmark; National Food Institute, Technical University of Denmark, Kgs. Lyngby, 2800, Denmark; National Food Institute, Technical University of Denmark, Kgs. Lyngby, 2800, Denmark; National Food Institute, Technical University of Denmark, Kgs. Lyngby, 2800, Denmark

**Keywords:** ulcerative colitis, probiotics, *E. coli* Nissle, microcontainer delivery, bacterial competition, micro device dosing system

## Abstract

For patients with ulcerative colitis (UC), administration of the probiotic *E. coli* Nissle (EcN) holds promise for alleviation of disease symptoms. The mechanisms are unclear, but it has been hypothesised that a capacity of the probiotic to outcompete potentially detrimental UC-associated *E. coli* strains plays an important role. However, this could previously not be confirmed in a mouse model of competition between EcN and two UC-associated strains, as reported by Petersen *et al*. 2011. In the present study, we re-evaluated the idea, hypothesising that delivery of EcN by a micro device dosing system (microcontainers), designed for delivery into the intestinal mucus, could support colonisation and confer a competition advantage compared to classical oral dosing. Six groups of mice were pre-colonised with one of two UC-associated *E. coli* strains followed by oral delivery of EcN, either in capsules containing microcontainers with freeze-dried EcN powder, capsules containing freeze-dried EcN powder, or as a fresh sucrose suspension. Co-colonisation between the probiotic and the disease-associated strains was observed regardless of dosing method, and no competition advantages linked to microcontainer delivery were identified within this setup. Other approaches are thus needed if the competitive capacity of EcN in the gut should be improved.

## Teaser

Delivery in microcontainers does not help a probiotic *E. coli* to establish in the mouse gut.

## Introduction

Ulcerative colitis (UC) is a relapsing, chronic disease affecting the colon with symptoms including bloody diarrhoea and abdominal pain. The aetiology is not yet well understood, although a complex interplay between genetics, environmental factors, abnormal immune responses and the gut microbiota is involved (Kobayashi et al. 2020). The presence of specific *E. coli* strains from the B2 and D phylogenetic groups is linked to UC (Kotlowski et al. 2007, Petersen et al. 2009), and it has been hypothesised that eradication of these strains may alleviate the disease. Relevant strategies to achieve this include antibiotic treatment and administration of probiotic bacteria with the ability to replace the UC-associated *E. coli* strains in the diseased intestine.

One of the most promising probiotic strains for treatment of UC is *E. coli* Nissle (EcN), which has been found to be equally effective in preventing relapses as the widely used drug 5-aminosalicylic acid (5-ASA) (Kruis et al. 2004) and to be an effective and safe add-on treatment to 5-ASA in patients with active UC for induction of remission and clinical response (Park et al. 2022). In contrast, no beneficial effect of EcN as add-on treatment to Ciprofloxacin was found in UC patients with active disease (Petersen et al. 2014). In DSS-treated mice administration of EcN is reported to alleviate disease symptoms, changes in immune response, and microbial dysbiosis (Rodríguez-Nogales et al. 2018). The underlying beneficial mechanisms are suggested to involve the known abilities of EcN to produce microcins, to possess a superior ability to compete for iron, to make robust biofilms in the mucus layer, to stimulate defensin production by epithelial cells, to strengthen tight junctions, and to interact with the immune system directing the cytokine expression towards a more anti-inflammatory profile (Sassone-Corsi et al. 2016, Scaldaferri et al. 2016, Zhao et al. 2022). These features support probiotic activity and might lead to the ability of EcN to outcompete and replace UC-associated *E. coli* strains.

This aspect was previously studied by Petersen et al. 2011, who administered EcN to streptomycin-treated mice, pre-colonised with one of two different B2 *E. coli* strains isolated from patients with active UC (Petersen et al. 2011). However, EcN did not outcompete the two UC-associated *E. coli* strains in this experimental setup, but co-colonised with the potentially detrimental strains. In the present study, we, therefore, re-evaluated the idea with the hypothesis that targeted delivery of EcN to a permissive intestinal niche could promote colonisation and hereby increase the competition ability against UC-associated *E. coli* strains. To obtain such targeted delivery, we used a microscale delivery system, denoted microcontainers (Nielsen et al. 2016a), into which powdered drugs (Christfort et al. 2020, Kamguyan et al. 2022, Torp, Kamguyan et al. 2022) or probiotics (Kamguyan et al. 2021, Christfort, Polhaus et al. 2022) can be loaded. Microcontainers have been fabricated in various shapes and sizes from 60 to 450 µM (Abid et al. 2019, Dalskov Mosgaard et al. 2019, Christfort et al. 2020, Chang et al. 2023, Petersen et al. 2015), but in this study, cylindrical devices with an inner diameter of 236 ± 1 µm (mean ± SD) and height of 218 ± 1 µm (mean ± SD) was used. The microcontainers were sealed with a polymeric lid, which protected from the acidic environment in the stomach and facilitated targeted release in the distal small intestine when encountering a pH above 6 in the environment (Shimizu et al. 2021, Christfort, Milián-Guimerá et al. 2022). Prior observations suggest that microcontainers will spread in the intestine and embed into the mucus layer enabling a dispersed and sustained release of drugs (Nielsen et al. 2016b, Mazzoni et al. 2017). Therefore, we hypothesised that delivery of probiotic EcN into the mucus layer by dosage in microcontainers would provide the strain with a colonisation advantage compared to simple delivery into the lumen. Supporting this hypothesis, *E. coli* is known to reside in the mucus layer and obtain nutrients from here (Conway and Cohen 2015). Additionally, microcontainer-based delivery results in dispersed seeding of flocks of EcN into different niches, and in combination, these characteristics were hypothesised to increase chances for successful colonisation and competition with UC-associated *E. coli* strains. We evaluated this *in vivo* using streptomycin-treated mice, each pre-colonised with one of two UC-associated *E. coli* strains, and subsequently administered with EcN by three different dosing methods, namely (i) freeze dried EcN powder in microcontainers loaded into gelatine capsules, (ii) freeze dried EcN powder loaded directly into gelatine capsules, (iii) a fresh sucrose suspension of EcN made from an overnight culture (Fig. S1). Colonisation of EcN and the UC-associated *E. coli* strains were then followed to compare effects of the different dosing methods of EcN.

## Materials and methods

### Bacterial strains and media

The bacterial strains used in this study were identical to those used previously by Petersen et al. 2011 (Petersen et al. 2011). Two *E. coli* strains isolated from UC patients with active disease, designated EcUC1 and EcUC2, were used. The EcUC1 strain is a spontaneous streptomycin resistant mutant of the isolated *E. coli* with an inserted kanamycin resistant gene cassette (GMO), and EcUC2 is a strain naturally resistant to ampicillin, sulphamethoxazole, streptomycin, trimethoprim and cephalothin. The EcN strain used is a spontaneous streptomycin and rifampicin resistant mutant (Petersen et al. 2011).

In the original references, EcUC1 was named IBD1 or p7, and EcUC2 was named IBD2 or p25 (Petersen et al. 2009, 2011). All strains were routinely cultivated in Luria-Bertani (LB) media (SSI Diagnostica, Hillerød, Denmark) overnight at 37°C.

### Freeze-drying EcN

An overnight culture (OD_600_ = 0.76) of EcN was freeze-dried as follows: Bacterial pellets obtained from centrifugation (4000 rpm, 10 min.) of 120 mL culture were resuspended in 5 mL lyoprotective media containing 5% skim milk powder/5% mannitol/10% sucrose in miliQ water, w/v (Sigma-Aldrich, St. Louis, USA) and snap frozen in dry ice for 20 min. in a sterile freeze-drying flask (Holm&Halby, Brønby, Denmark). Freeze-drying was conducted overnight on a Telstar LYOQUEST -55 PLUS manifold tabletop freeze-drier (Holm&Halby, Brøndby, Denmark) with a condenser temperature of −55°C and a pressure of 0.3 mbar. The resulting freeze-dried product was ground to a fine powder in a sterile grinder. In total, 0.7 g of EcN powder was obtained with approximately 9.5·10^9^ CFU/g powder.

### Microcontainer fabrication, loading and coating

The microcontainers used in this study are microfabricated cylindrical devices designed for unidirectional release and have an inner diameter of 236 ± 1 µm (mean ± SD) and inner height of 218 ± 1 µm (mean ± SD). They were fabricated from SU8, a negative epoxy photoresist (SU-82035, 2075 and SU-8 developer, micro resist technology GmbH, Berlin, Germany) through a two-step photolithographic process on silicon wafers coated with a layer of 5 nm Ti and 20 nm Au to enable easy release of the microcontainers. Afterwards, the silicon wafers were cut into chips containing 625 microcontainers each. The fabrication procedure was previously described ((Kamguyan et al. 2021).

Microcontainers were manually loaded with freeze-dried EcN powder by applying a shadow mask that covers the spaces between the single microcontainers on a chip and using a brush to push the powder inside the microcontainers. After removal of the mask, the microcontainers were spray coated with 1% w/v Eudragit® L100, soluble above pH 6 (Evonik, Essen, Germany) in an isopropanol solution and 5% w/w (in relation to the polymer) dibutyl sebacate using an ultrasonic spray coater (Exactacoat system, Sono-Tek, Milton, NY, USA). The Accumist nozzle was operating at 120 kHz and each chip was coated with 30 loops, while laying on a plate heated to 40°C to ensure proper evaporation of the solvent. Both loading and coating was evaluated in a TM3030Plus tabletop scanning electron microscope (SEM, Hitachi High Technologies Europe GmbH, Krefeld, Germany).

### Dosing strategies for EcN

EcN was prepared in three different dosing systems: (i) Microcontainers in capsules. Microcontainers were loaded with freeze-dried EcN powder and coated with Eudragit® L100 (Evonik, Essen, Germany) using a spray-coater (Exactacoat system, Sono-Tek, Milton, NY, USA) as described earlier. Microcontainers were filled into gelatine capsules size M (Torpac, NJ, USA), which disintegrate in the stomach, resulting in a dose of EcN of app. 3·10^5^ CFU/mouse. (ii) Powder in capsules. Freeze-dried EcN powder were filled directly into gelatine capsules size M (Torpac, NJ, USA), which disintegrate in the stomach, resulting in a dose of EcN of app. 5·10^6^ CFU/mouse. (iii) Sucrose suspension. An inoculum of EcN was prepared from overnight cultures, with bacterial pellets resuspended in 20% sucrose (w/v) (Sigma-Aldrich, St. Louis, USA), resulting in a dose of EcN of app. 4·10^8^ CFU/mouse. The doses were estimated, taking losses during freeze-drying, storage and handling procedures into account.

### Animals and experimental setup

Animal experiments were carried out under approval by the Animal Welfare Committee, license number 2015 − 15 − 0201 − 00553 by trained and skilled personal at the animal facility at the National Food Institute, Technical University of Denmark. About 48 female NMRI mice, 6–8 weeks old (Taconic, Ejby, Denmark) were housed two mice in each cage in controlled, ventilated cabinets (ScanTainer, Scanbur, Karlslunde, Denmark) with *ad libitum* access to feed and drinking water containing 5 g/L streptomycin sulphate from 3 days prior to experimental start and throughout the entire study period (18 days). The mice were randomised into 6 groups with 8 mice in each. Each mouse was first pre-colonised with one of two UC-associated *E. coli* strains, denoted EcUC1 and EcUC2, and afterwards EcN was administered by one of three dosing methods. Group 1: EcUC1 and microcontainers with EcN loaded to capsules, Group 2: EcUC2 and microcontainers with EcN loaded to capsules, Group 3: EcUC1 and EcN powder in capsules, Group 4: EcUC2 and EcN powder in capsules, Group 5: EcUC1 and a sucrose suspension of EcN, and Group 6: EcUC2 and a sucrose suspension of EcN (see Supplementary Fig. 1).

**Figure 1.**
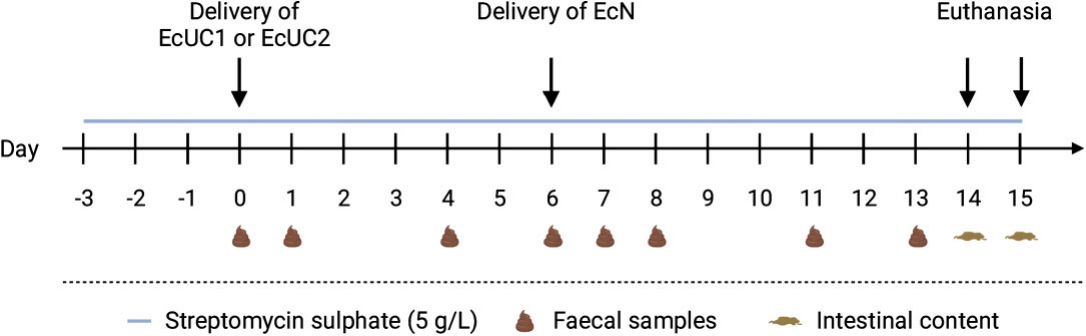
Timeline for study. Created with bioRender.com.

On Day 0, all animals were dosed once by gavage with an inoculum of either EcUC1 or EcUC2 (app. 3·10^9^ CFU/mouse), prepared from overnight cultures, with bacterial pellets resuspended in 20% sucrose (w/v) (Sigma-Aldrich, St. Louis, USA). On Day 6, a single dose of EcN was administered by gavage to all animals, using the three described dosing systems. The animals were euthanized on Days 14 and 15, half of each group each day (Fig. 1).

### CFU counting in faeces and intestinal content

In line with previous observations (Petersen et al. 2011), we observed that LB agar plates containing 100 mg/L streptomycin (STR) + 25 mg/L kanamycin (KAN) were selective for EcUC1, and that plates with 100 mg/L streptomycin (STR) + 100 mg/L rifampicin (RIF) were selective for EcN. In order to select for EcUC2, we increased the ampicillin content from 50 mg/L suggested by Petersen et al. to 100 mg/L, ending up using plates with 100 mg/L streptomycin (STR) + 100 mg/L ampicillin (AMP).

Additionally, it was tested whether faeces from NMRI mice inherently contains bacteria, which can grow on the selective antibiotic agar plates used in the study. No growth was observed.

During the study, faecal samples were collected from all mice on Days 0, 1, 4, 6, 7, 8, 11, and 13 (Fig. 1). All faecal pellets were weighed, diluted 10x in sterile 0.9% NaCl and homogenised. Ten-fold serial dilutions of all samples were spot-plated (10 µL) on relevant selective LB agar plates (SSI Diagnostica, Hillerød, Denmark) as follows: Faecal samples from animals receiving EcUC1 were plated on plates selective for the EcUC1 strain (LB + STR + KAN) and EcN (LB + STR + RIF), whereas samples from animals dosed with EcUC2 were plated on plates selective for the EcUC2 strain (LB + STR + AMP) and EcN (LB + STR + RIF). All plates were incubated aerobically over night at 37 ˚C and the colony forming units (CFU) were counted.

On the dissection days (Days 14 and 15), contents from the ileum, caecum and colon were collected. The samples were handled following the same procedure as used for the faecal pellets.

### Data analysis and statistics

From CFU counts, CFU/g faeces and CFU/g intestinal content were calculated and log_10_-transformed. Statistical analysis and visualisation of data was performed in R (R Core Team 2023), using RStudio and the packages tidyverse (Wickham et al. 2019), ggpubr (Kassambara 2023a), and rstatix (Kassambara 2023b). Differences in EcN CFU/g on Day 7 and Day 8 were analysed by a one-way ANOVA test with FDR adjustment, followed by pairwise comparison between delivery groups of significant interactions using a Tukey test with FDR adjustment. Prior to the analysis, parametric assumptions were tested and found to be met.

Comparison of EcUC1/EcUC2 and EcN CFU/g in the intestinal compartments, ileum, caecum and colon by delivery was performed by a non-parametric Kruskal-Wallis test with FDR adjustment followed by a pairwise Wilcoxon test with FDR adjustment for the significant interactions identified. Prior to the analysis, parametric assumptions were tested, but several extreme outliers were identified and the assumption of homogeneity of variance was violated. Differences in ratios between EcN and EcUC were analysed by a Kruskal Wallis test with FDR adjustment, followed by a pairwise Wilcoxon test with FDR adjustment. The parametric assumption of normality of data was found to be violated.

Statistical significance was set to *P* < 0.05, where * = *P* < 0.05, ** = *P* < 0.01, *** = *P* < 0.001, ^****^ = *P* < 0.0001.

## Results and discussion

**Figure 2.**
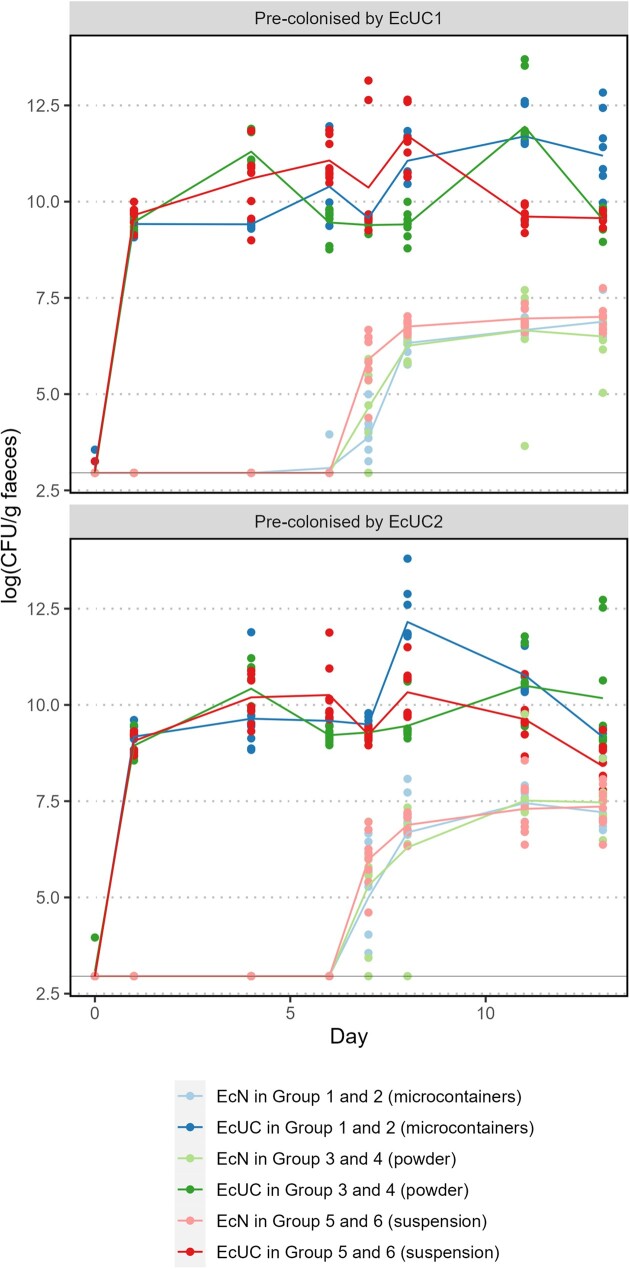
Log(CFU/g faeces) in animals pre-colonised by EcUC1 or EcUC2 on Day 0, followed by dosing on Day 6 of EcN in Group 1 and 2 by microcontainers (blue), in Group 3 and 4 by powder (green) and in Group 5 and 6 by a suspension (red). Light colours represent EcN log(CFU/g), whereas dark colours are EcUC log(CFU/g). Results are shown as individual measures with curves representing the means (N = 8). The detection limit is marked with a line.

To evaluate whether microcontainer-based delivery of the probiotic EcN results in an increased colonisation and competition ability compared to simple oral delivery strategies, colonisation levels of the two UC-associated *E. coli* strains, EcUC1 and EcUC2, and EcN were followed for two weeks. For each animal CFU/g faeces of EcUC1/EcUC2 and EcN was calculated for Days 0, 1, 4, 6, 7, 8, 11, and 13 (Fig. 2).

**Figure 3.**
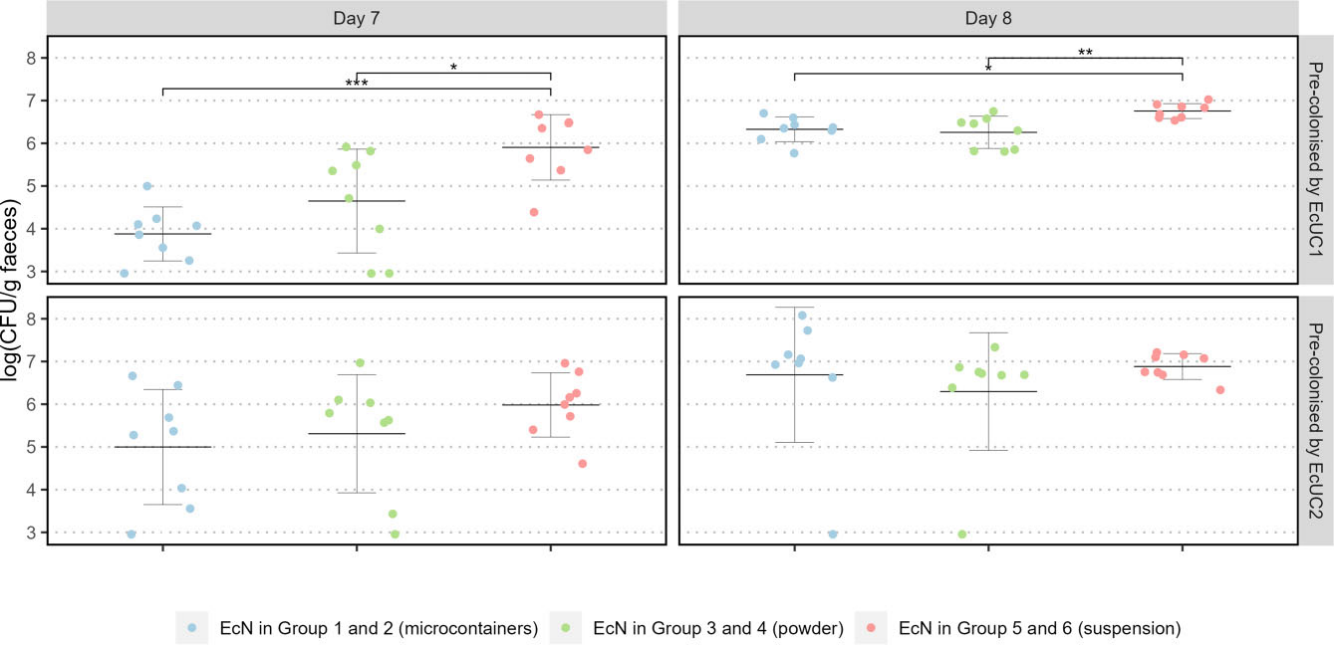
Log(CFU/g faeces) for EcN on Day 7 and Day 8 after dosing in Group 1 and 2 by microcontainers (blue), in Group 3 and 4 by powder (green) and in Group 5 and 6 by a suspension (red). Sample values are shown as individual measures with mean and SD (N = 8). * = *P* < 0.05, ** = *P* < 0.01, *** = *P* < 0.001.

Successful pre-colonisation by EcUC1 or EcUC2 occurred in all groups (Fig. 2). After administration of EcN on Day 6, the incoming probiotic strain co-colonised with the UC-associated strains and reached a stable level several log-values below these strains. Co-colonisation was observed regardless of the dosing method for EcN. To reveal differences between the dosing methods occurring immediately after probiotic administration, we specifically investigated EcN levels during the first two days after delivery, Days 7 and 8 (Fig. 3).

In mice pre-colonised by EcUC1, significantly higher levels of faecal EcN were found when delivered in a sucrose suspension compared to powder on both Day 7 (*P* = 0.030) and Day 8 (*P* = 0.0072) and compared to microcontainers on both Day 7 (*P* = 0.00059) and Day 8 (*P* = 0.022) (Fig. 3). These inter-group differences may reflect differences in colonisation ability between dosing methods, but it should be noted that initial dosing levels were not identical, and this may explain some or even all the differences observed. Group 1 and 2 (microcontainers) were dosed app. 3·10^5^ CFU/mouse, Group 3 and 4 (powder) got app. 5·10^6^ CFU/mouse, while Group 5 and 6 (suspension) got app. 4·10^8^ CFU/mouse. The lower doses for Group 1 and 2 resulted from unexpected viability losses of EcN during microcontainer coating and storage. According to OD measurements of the overnight culture administered to Group 5 and 6, the dose level was equal to that given to Group 3 and 4. However, subsequent CFU counting revealed he differences listed above.

**Figure 4.**
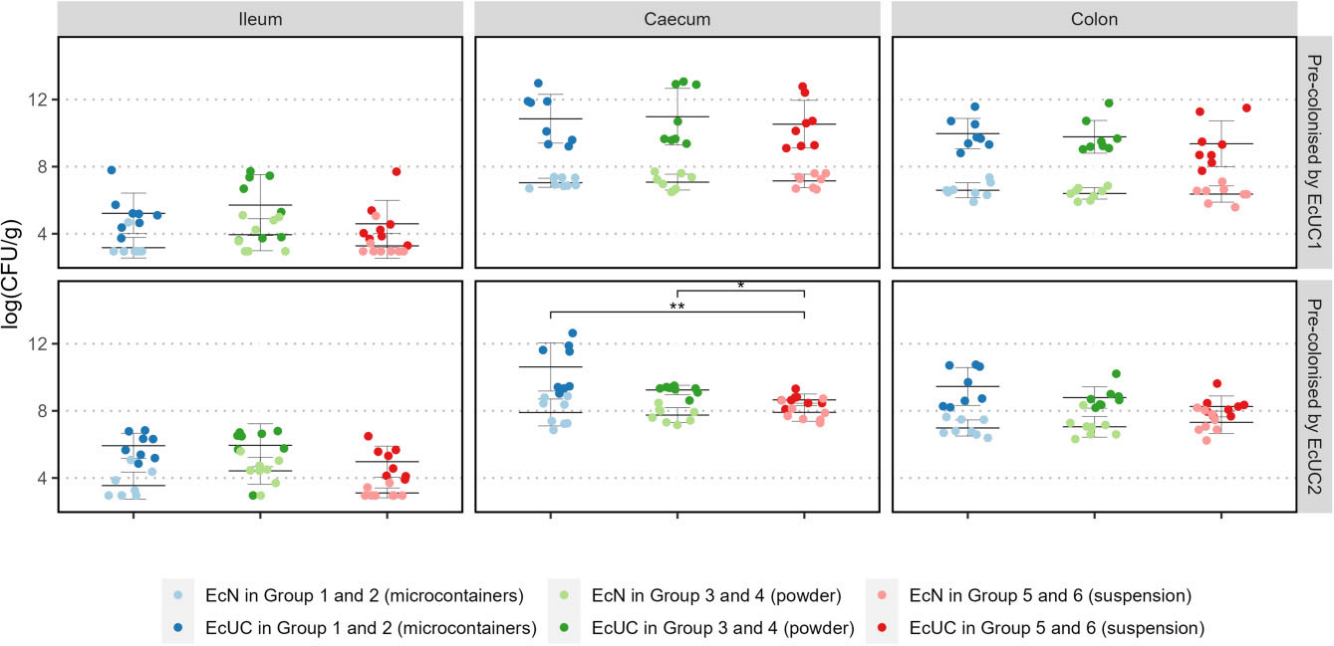
Log(CFU/g) on dissection Days 14 and 15 for ileum, caecum and colon. Dosing in Group 1 and 2 by microcontainers (blue), in Group 3 and 4 by powder (green) and in Group 5 and 6 by a suspension (red). Sample values are shown as individual measures with mean and SD (N = 8). * = *P* < 0.05, ** = *P* < 0.01.

On dissection (Days 14 and 15), concentrations of EcUC1/EcUC2 and EcN in ileum, caecum and colon were assessed (Fig. 4). In line with the findings in faeces (Fig. 2), EcUC1/EcUC2 were more abundant than EcN in all three intestinal compartments. The same compartments were colonised by all strains, potentially allowing competition between them. Highest levels of all strains occurred in the caecum and colon, whereas lower concentrations were found in ileum. In caecum for animals pre-colonised by EcUC2, the level of this disease-associated strain was significantly lower in Group 6 (suspension) compared to the same strain in Group 2 (microcontainers) (*P* = 0.003) and Group 4 (powder) (*P* = 0.015).

The EcUC strains and EcN are all members of the B2 phylogenetic group (Petersen et al. 2011), and hence share characteristics, but possess different virulence factors. The observed co-colonisation suggests that the EcUC strains and EcN occupy different nutritional and/or spatial niches, since directly competing isogenic *E. coli* strains do not co-colonise in streptomycin-treated mice (Leatham et al. 2009).

To account for differences between animals, ratios between EcN and the UC-associated *E. coli* strains within each individual mouse were calculated (see Supplementary Fig. 2). No consistent pattern was observed, but the ratio was significantly different between groups at several time points. Ratios tended to be highest in groups, which received EcN as freeze-dried powder or in a fresh suspension. Thus, microcontainer-based delivery of EcN did not confer any advantage to the probiotic strain, which means that the initial hypothesis could not be confirmed. A number of factors may explain the lacking effect of microcontainer-based delivery in the given mouse model. First, the mucus layer of the mouse is app. 150 µm (Johansson et al. 2011), and thus relatively thin compared to the size of the microcontainers, which measure 254 ± 2 µm (mean ± SD) in total height, and therefore targeted delivery into the mucus may not be optimal. Second, *E. coli* strains are known to be resistant to the acidic gastric environment (Foster 2004, Pienaar et al. 2019, Xu et al. 2020), and the protection offered by the microcontainers, is then not conferring any extra advantage for EcN to survive through the stomach, as confirmed by our results for capsule and suspension-based dosing strategies (Fig. 2). We suggest that microcontainer-based delivery may be feasible for fragile next-generation probiotics such as *F. prausnitzii* (Torp, Bahl et al. 2022). However, preparation methods including loading and coating of microcontainers are not suitable for handling oxygen-sensitive strains, and alternative approaches remain to be developed.

## Supplementary Material

fnad110_Supplemental_File
