# Supplementary material for: Delivery of E. coli Nissle to the mouse gut by mucoadhesive microcontainers does not improve its competitive ability against strains linked to ulcerative colitis
Source: FEMS Microbiol Lett. 2023 Oct 20;370:fnad110. doi: 10.1093/femsle/fnad110 (PMC10612143; doi:10.1093/femsle/fnad110)
Supplement: fnad110_Supplemental_File [file fnad110_supplemental_file.pdf]

Delivery of *E. coli* Nissle to the mouse gut by mucoadhesive microcontainers does not improve its competitive ability against strains linked to ulcerative colitis

Supplementary Materials

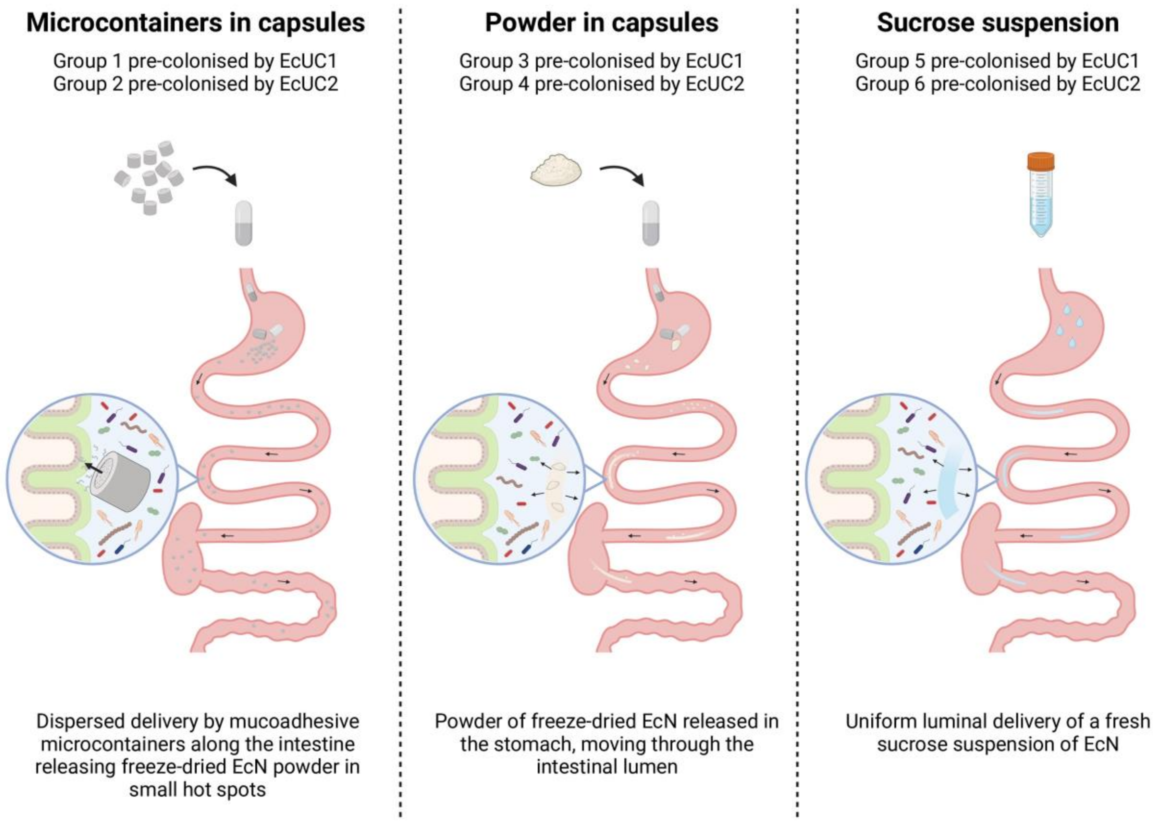

Supplementary Figure 1. *Delivery methods for EcN: Group 1 and 2 (microcontainers), Group 3 and 4 (powder) and Group 5 and 6 (suspension). Created with bioRender.com.*

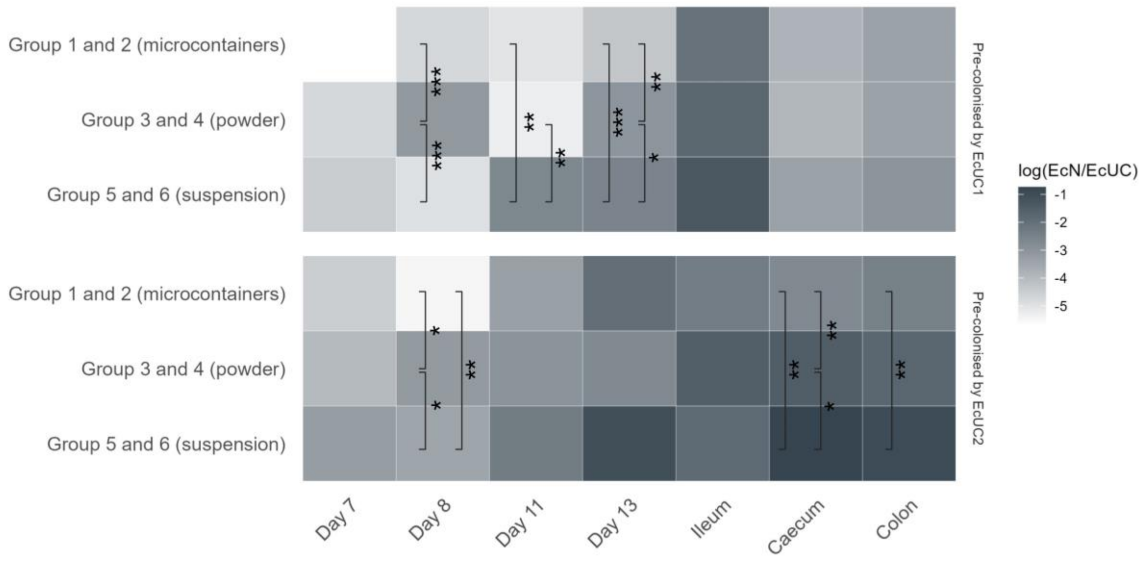

Supplementary Figure 2. *Ratio between CFU counts for EcN and EcUC (Log(EcN/EcUC)). The higher the ratio, the higher proportion of EcN compared to EcUC is found. The ratio is calculated for each mouse, represented as means in the heatmap (N=8). * = $p < 0.05$, ** = $p < 0.01$, *** = $p < 0.001$, **** = $p < 0.0001$. Significant findings are marked in the figure using Biorender.com.*
